# Will Public Health Emergencies Affect Compensatory Consumption Behavior? Evidence from Emotional Eating Perspective

**DOI:** 10.3390/foods13223571

**Published:** 2024-11-08

**Authors:** Yi-Fei Wang, Kai-Hua Wang

**Affiliations:** 1 School of Business, Beijing Normal University, Beijing 100875, China; 13206458218@163.com; 2 School of Economics, Qingdao University, Qingdao 266100, China

**Keywords:** consumer behavior, COVID-19 pandemic, compensatory consumption, emotional eating, social media sentiment analysis

## Abstract

This research examines the correlation between the COVID-19 pandemic and the desire to engage in compensatory consuming behaviors, specifically emphasizing emotional eating as a psychological coping strategy, particularly with respect to snacks and sweets. Conducting sentiment analysis by using a Natural Language Processing (NLP) method on posts from Sina Weibo, a leading Chinese social media platform, the research identifies three distinct phases of consumer behavior during the pandemic: anxiety, escapism, and compensatory periods. These stages are marked by varying degrees of emotional eating tendencies, illustrating a psychological trajectory from initial shock to seeking comfort through food as a means of regaining a sense of normalcy and control. The analysis reveals a notable increase in posts expressing a desire for compensatory consumption of snacks and sweets in 2020 compared to 2019, indicating a significant shift towards emotional eating amid the pandemic. This shift reflects the broader psychological impacts of the crisis, offering insights into consumer behavior and the role of digital platforms in capturing public sentiment during global crises. The findings have implications for policymakers, health professionals, and the food industry, suggesting the need for strategies to address the psychological and behavioral effects of natural disasters.

## 1. Introduction

In 2020, the outbreak of the coronavirus disease (COVID-19) had a tremendous impact on the global economy and people’s personal safety [1,2,3]. Governmental organizations tried to restrict the virus since it spread quickly by enforcing quarantine and protective measures or policies of social distancing and lockdowns [4,5]. During this period of history, most governments implemented lockdowns, curfews, and orders to remain at home. There were 1.7 billion individuals under lockdown globally as of 26 March [6], and by 7 April, there were 3.9 billion, or more than half of the world’s population, under lockdown [7]. As of 7 March 2020, the World Health Organization (WHO) reported that the global number of confirmed cases of COVID-19 had surpassed 100,000, with an escalating severity leading to a significant loss of life due to infections from the novel coronavirus. By 11 March, the WHO officially declared COVID-19 a pandemic [8]. Apart from the severe impact on human physical health, recent studies also have confirmed that the COVID-19 pandemic has had a significant effect on the mental health of individuals worldwide [9,10], which was very likely to cause dramatic changes in human behavior. A report by the WHO highlighted that in the first year of the pandemic, there was a 25% increase in the global prevalence of anxiety and depression [11]. The increase in mental health issues was attributed to several factors, including social isolation, financial worries or losses, fear of infection, and the stress and grief of coping with the loss of loved ones [12]. When a public health catastrophe strikes so quickly, individuals often feel like they have little control over their own lives [13], which can lead to the unconscious release of stress. The phrase “compensatory consumption” was first used by researchers to describe any acquisition, application, or consumption of goods or services driven by a desire to make up for or lessen such a shortfall [14,15,16]. One crucial and convenient way to compensate for the gap between an ideal situation in mind and the realistic situation of compensatory consumption is by consuming food [17]. More specifically, mitigating stress and depression or other low emotional states through consuming food is called emotional eating [18]. In this study, eating snacks and sweets, two common forms of compensatory consumption and emotional eating [16,19], are regarded as methods of psychological compensation. The purpose of this study is to determine if the COVID-19 pandemic threat has influenced the intentions of compensatory consumption, particularly regarding emotional eating.

Having experienced all stages of this pandemic in a concentrated time period, being the first country that was severely affected, China not only launched strict lockdown policies in response to this but was one of a small number of countries that chased an elimination strategy [20,21], sustaining zero or low case numbers over the long term [22]. From 23 January to 25 January 2020, Chinese provinces and autonomous areas sequentially declared a state of emergency, enforcing an unprecedented complete lockdown and isolation [23,24], affecting approximately 1.2 billion Chinese residents [25]. Chinese families were forced to live in close quarters and adhere to stringent social distancing guidelines outside of the house as a result of experiencing a quarantine and prolonged home confinement. This posed a danger to the psychological health of residents in the most impacted parts of China [12,26,27]. This makes China a typical case for studying compensatory consumption psychology [25].

This article captures microblog text data from 20 January to 30 March for the years 2019 and 2020 by using keywords “want to eat snack” and “want to eat sweet” on the Sina Weibo platform—the biggest public information dissemination platform in China—to analyze the compensatory eating intention during the pandemic in China. This research aims to investigate the emotional eating sentiment during the COVID-19 pandemic, to reflect and uncover whether people were truly inclined to conduct compensatory consumption in this period. We address the dynamic shifts in the emotional eating tendencies of snacks and sweets during the focused COVID-19 period in China, seeing them as an analytical focus and breakthrough points.

Based on the Natural Language Processing (NLP) method, this present paper conducted a sentiment analysis on these captured microblogs, and we obtained the following results: First, our research demonstrates that the COVID-19 epidemic had a major impact on consumers’ psychological states, leading to an increased tendency towards eating snacks and sweets compared to 2019, especially significant at the end of concentrated outbreak period. More importantly, three distinct stages are identified in consumer behavior during the pandemic: the anxiety period, the escapism period, and the compensatory period. Each stage corresponds with specific changes in the public’s emotional state and compensatory eating patterns.

The marginal contributions of this paper can be summarized below. First, with reference to the data collection, which is the core part of compensatory consumption research, the predominant data collecting approach in previous compensatory consumption studies revolved around self-administered questionnaires and surveys. There is limited literature that has studied this phenomenon using real-time data instead of self-reported data. This paper innovatively deviates from this norm by employing a novel methodology of gathering lexical data from social media platforms. This innovative approach aims to examine whether individuals, when confronted with the threatening environment of the COVID-19 pandemic, exhibited motivations for engaging in compensatory eating. It provides valuable insights into the real-time emotional states of consumers offering a novel perspective in understanding the dynamics of compensatory consumption. Additionally, this study combines the emotional eating motivations of people during the pandemic with the three stages of compensatory consumption, namely the anxiety period, the escapism period, and the compensatory period, which explain the real-time dynamic process of compensatory eating intention during the COVID-19 pandemic. This provides empirical support for future research on people’s compensatory consumption and emotional eating motivations in response to public health emergencies or natural disasters.

The remainder of this essay is structured as follows: Section 2 includes relevant literature for this investigation. Section 3 discloses the fundamental theories used in this paper. Section 4 introduces the methods of NLP. Section 5 describes how data were collected and processed. Section 6 shows the empirical results. Section 7 discusses the results. Section 8 summarizes the study.

## 2. Literature Review

### 2.1. Eating Behavior Changes During COVID-19 Pandemic

Numerous studies have shown that people’s eating habits have changed during the COVID-19 pandemic. In Italy, a survey noted that nearly half the residents felt they gained weight during the early COVID-19 lockdown [28]. Similarly, an international study across eight countries found that lockdowns caused shifts towards less healthy eating habits, influenced by anxiety or boredom [29], highlighting a global trend in changing dietary patterns during this period. During Italy’s lockdown, nearly half of the participants ate more, with 19.5% gaining weight from increased anxiety and consuming more comfort foods like desserts and salty snacks [30]. In the U.S., household spending dropped by 25–30%, except for a rise in groceries and food delivery, reflecting shifts in consumer behavior [31]. A study during Egypt’s COVID-19 lockdown also showed that dietary patterns shifted, with an uptick in unhealthy food consumption and changes in physical activity and weight across age and BMI groups [32]. A study in Riyadh during COVID-19 noted more home-cooked meals and changes in food quality, highlighting the importance of promoting healthy eating during a pandemic [33].

### 2.2. Compensatory Consumption and Emotional Eating

The phrase “compensatory consumption” refers to a broad range of consumer intents and behavioral reactions brought on by various perceived deficiencies, needs, and wants that are not immediately met. Considering this, they receive compensation via other channels [25,34]. This behavior ranges from private, repetitive buying that may lead to negative outcomes, to satisfying public, product-focused interactions [35]. Different types of compensatory consumption may involve various products or services. Researchers have found that when an individual feels that their appearance is compromised, they often show an interest in purchasing clothes that enhance their appearance [36]. Wicklund and Gollwitzer [37] observed that MBA students lacking objective business success indicators (like a high GPA or multiple job offers) often displayed symbolic indicators such as wearing expensive attire and watches. This compensatory behavior, however, did not alter their actual academic performance. For another instance, researchers discovered that people tried to regain control by selecting goods with symbolic boundaries (framed vs. unframed paintings) when they felt as if they had lost control [38]. Sports enthusiasts ate meals higher in calories and saturated fat when their hometown team lost a game as opposed to when they won, according to research by Cornil and Chandon [39]. In addition, escapist films or television shows may be watched on a “binge watch” basis by those who want to escape from their own attention [40]. When facing a discrepancy between the real self and the ideal self, choosing to purchase compensatory food can often be considered a form of avoidance and compensatory consumption behavior [15]. This method of consuming in response to negative emotions is also modernly referred to as retail therapy and emotional eating [41]. 

Emotional eating, often referred to as stress eating, involves eating in reaction to emotional states, particularly negative ones, rather than from actual hunger or physical need [18]. The idea of emotional eating was first introduced in a 1999 research study by Conner et al. [42] that looked at the connection between emotions and snacking. It may also be thought of as a means of reducing stress, despair, and other depressive emotional states. Emotional eating has also been shown to be associated with decreased positive effects and greater negative effects. However, emotional eating does not seem to control effects well [43], and the combination of this ineffectiveness with persistent negative behaviors could worsen emotional regulation with food, potentially leading to an eating addiction [18]. Eating as a reaction to emotional states can sometimes cause reverse effects, as evidenced by research linking emotional eating with an increase in weight gain [44,45], depression [46,47], and both depression and weight gain in turn, which makes a positive difference on emotional eating [48]. Moreover, emotional eating can also cause immediate distress, such as inducing feelings of guilt [49], with evidence from the Christchurch earthquake also showing people turn to overeating in response to disaster-related stress [50].

### 2.3. Emotional Eating Research During the COVID-19 Pandemic

Many occurrences, like the COVID-19 pandemic and SARS, have the potential to increase population death rates while simultaneously causing a loss of personal liberty because of their abrupt and unpredictable character [13]. This kind of reduction in personal freedom during the COVID-19 pandemic can trigger compensatory consumption behaviors [25]. Basically, and essentially, consumers often gravitate towards products that symbolically counterbalance perceived threats, which could manifest in a range of compensatory buying behaviors [15]. This is because significant infectious diseases pose a threat to individuals’ survival and can heighten their inclination to pursue risk and diversify their choice of commodities, which may amplify their indulgent consumption tendencies [51,52].

Especially, regarding eating due to negative emotions, a research study conducted during the COVID-19 epidemic on young Saudi women found that over half of them related emotional eating during stressful times to an increase in unhealthy food consumption, including sugar and fat, more meals, and frequent fast-food consumption [53]. An Egypt-based research study conducted in a similar way also showed that lockdown-related stress contributed to increased emotional eating behaviors during this pandemic lockdown [54]. Additionally, a Polish study conducted during COVID-19 discovered a significant relationship between stress and effect regulation and emotional overeating, indicating that eating may serve as a coping mechanism for negative emotions related to the pandemic. If food becomes the primary means of regulating emotions, this could potentially result in eating disorders [55]. Additional research found that fear of COVID-19 and associated depressive symptoms acted as a mediator between increased emotional eating and negative emotional reactivity, which was substantially and directly correlated with the former [56,57]. Further evidence of social media’s function in exacerbating anxiety and eating behavior comes from the fact that 48% of Chinese respondents during COVID-19 lockdowns reported higher emotional overeating and high-calorie cravings, which were connected to prolonged exposure to social media, neuroticism, and anxiety [58].

According to Costa et al. [59], emotional eating has also been linked to a decrease in vegetable intake and an increase in the consumption of sweets and desserts. Furthermore, studies have shown that emotional overeating increases the consumption of meals rich in sugar [60]. Hepworth et al. [61] have shown that negative mood states or chronic stress might also increase subjective hunger, food cravings, and preferences for high-calorie foods (such as chocolate and sweets). Concerns about one’s health, psychological anguish, and personal finances were risk factors for the development of emotional eating [62]. Students who claimed that they often eat more during stressful times consumed more ice cream than those who reported eating less [63]. The ego-threatening stressor caused emotional eaters to consume more chocolate than in the control condition, but there were no changes in chocolate consumption between the cognitive stressor and control [64]. Further, Jansen et al. [65], Bakaloudi et al. [66], and Curtin et al. [67] also verified notable snack habits during the COVID-19 pandemic. In conclusion, we are inclined to choose sweets and snacks as the proxies of emotional eating.

## 3. Theory Basement

The release theory and compensatory theory served as the foundation for our investigation. According to the release theory, people will eventually let go of repressed actions, ideas, and feelings to mitigate their pressure [25,68]. The onset of COVID-19 disrupted people’s established rhythms of life. Spatial isolation, restrictive measures, daily updates on COVID-19 case numbers and fatalities, and economic downturn imposed significant physical and psychological stress on individuals. People were forced to repress their regular everyday habits, thoughts, and feelings when faced with a danger to their lives. However, within the context of the pandemic, finding a way to release this pent-up stress became essential for allowing their exhausted and anxious minds and bodies to relax. The concept of compensatory consumption refers to the desire to purchase, use, or consume products or services as a means of compensating for or reducing the sense of loss of control and lack of self-control [35,69].

The compensation problem is theoretically and philosophically owed to Adler [70]. According to Adler, the root of all psychological phenomena is a sense of inadequacy that motivates an individual to rise above negative experiences and go over them [70]. Caplovitz [71] established the notion of compensatory spending, pointing out that those with lesser earnings might have greater costs in the U.S. Gronmo [72] asserts that people often try to make up for any shortcomings in their life. However, by turning to workarounds, these actions relieve people’s symptoms without addressing the underlying cause [73]. 

To be more precise, following Rothbaum et al. [74], Cutright [38], and Zhang et al. [25], this paper also classifies compensatory consumption into three distinct phases. The first phase, wherein people struggle with worry and increased stress brought on by the lack of control and life-threatening situations, is called the anxiety period. An intense desire to reduce tension and anxiety characterizes the second stage, which is called the escapism period. In the third stage, consumers use items that symbolically address their emotions of tension, worry, and helplessness to reduce their purchasing habits and achieve an inner sense of serenity. This period is called the compensatory period. Building upon this foundation, this study aims to empirically examine whether people’s compensatory consumption motivations, particularly in the context of compensatory eating motives, have shown an increase during the pandemic.

## 4. Method Specifications

In this study, we utilized Natural Language Processing (NLP) technology to conduct sentiment analysis on the content of microblogs. NLP, a branch of linguistics, computer science, and artificial intelligence, focuses on the interaction between computer systems and human language. It focuses on computer programming techniques for handling and evaluating large amounts of natural language input. Recently, NLP has been highlighted as a highly discussed technology and has seen remarkable advancements, particularly due to deep learning. Deep learning, a subset of machine learning, is inspired by the brain’s structure and function, using algorithms known as artificial neural networks. For our emotional analysis, we chose Baidu’s AI-based natural language processing platform, which employs a dictionary-based approach. We used Baidu’s application programming interface (API) to extract emotional tendencies from microblogs, categorizing them into positive, neutral, and negative sentiments. Another part of NLP, sentiment analysis, is a key tool in understanding public opinion on social media and a complex process that involves various techniques, such as opinion mining and online textual data processing [25,75]. In recent years, one of the growing trends is that with the growth of Internet technology and mobile social media platforms, people are increasingly sharing their thoughts and feelings on the Internet and social platforms when they experience certain events. Numerous empirical research efforts have been made by scholars to comprehend the emotional responses of users to social media content [76,77], utilizing the gathering and examination of online textual data from social media users [78,79,80]. 

By using NLP technology, social networking sentiment analysis has been conducted widely in previous research [81,82,83]. For instance, it has been conducted in research using Twitter comment data to conduct in-depth sentiment analysis on a substantial volume of Twitter postings [84,85,86]; 71% of Twitter users claim to use the platform as a news source, according to a study, demonstrating the enormous influence of popular social media on public opinion formation [87]. Researchers have also examined Sina Weibo microblogs for film reviews, and as a result, they have developed an emotional language exclusive to the film industry [25,88,89]. And some research is based on Facebook textual data [90,91]. Using assessments from catering platforms, researchers have also looked at patrons’ emotional preferences at different restaurants [92]. The emotional categorization method for hotels and tourist sites has been built by researchers using rating data derived from an online travel platform in the field of eating research [93].

Sentiment analysis of the COVID-19 pandemic has gained attention in recent years as a means of evaluating social media’s performance for pandemic-related sentiment measures and gaining an understanding of attitudes and viewpoints regarding the COVID-19 vaccination in Italy [94]. Scholars have classified pertinent research in the realm of social media data mining within the framework of COVID-19 into six primary categories, including early warning and detection, human movement tracking, and other areas [87]. Researchers assessed the impact of COVID-19 cases on the emotions of the Greek population by evaluating real-time tweets on Twitter [95]. Imran et al. [96] presented a comprehensive dataset including 2 billion multilingual Twitter tweets, which has been used for the purpose of examining and comprehending the ramifications of the COVID-19 pandemic on individuals’ health, livelihoods, and social welfare on a worldwide level.

Nevertheless, in spite of the substantial limitations imposed on individuals’ autonomy within the COVID-19 epidemic, there have been very few studies using sentiment analysis methods to investigate changes in people’s compensatory consumption motivations. Therefore, this study will focus on researching this issue. 

## 5. Data Collection and Processing

For the convenience of data retrieval and analysis, this research exclusively concentrated on Sina Weibo, which is a cost-free social media network in China that facilitates real-time communication and engagement among registered users via the content they provide. This platform has become the prevailing and widely used Chinese microblogging medium [58]. In this study, data from Sina Weibo were retrieved using Python 3.x software. In order to eliminate advertisements for sweets and snacks, sharing of consumption, and other irrelevant topics on microblogging, this study used the keywords “want to eat snacks” and “want to eat sweets” to filter for relevant microblog content. This enabled a more accurate evaluation of consumer sentiments towards these two categories of compensatory consumption food. In terms of the selection of time periods, this study focuses on the duration between 20 January and 30 March for both the years 2019 and 2020.

For the year 2020, a significant development occurred on 20 January when the National Health Commission of China announced that COVID-19 would be classified as a Class B infectious disease, but would be managed with Class A infectious disease control measures. The Commission also reported human-to-human transmission of the virus. This announcement signaled a nationwide tightening of prevention and control measures and simultaneously stirred considerable panic and anxiety among the public. Regarding the timeframe of the data selected for this study, by the end of March, the number of new domestic cases in China had gradually decreased to below 100. By 30 March, the number of new cases within China had dropped to zero, with all new cases being imported. China implemented immediate quarantine measures for these imported cases, making the situation relatively controllable. To maintain consistency in the time variable and to study the impact of the COVID-19 pandemic on compensatory consumption and emotional eating, this research aligns the time periods selected for 2019 with those chosen for 2020. 

In this research, we utilized a Python-based program to extract all Weibo posts from 20 January to 30 March for the years 2019 and 2020, which served as our primary dataset. A total of 43,012 entries were collected for the year 2020, among which 29,598 posts were retrieved using the search term “want to eat snacks”, and 13,414 posts were gathered using “want to eat sweets”. For the year 2019, the total number of entries amounted to 23,228, with 14,714 posts acquired through the search query “want to eat snacks” and 8514 posts via “want to eat sweets”. This study meticulously organized data comprising user IDs, content of the Weibo posts, time of publication, and location of posting. Table 1 presents some samples for illustrative purposes.

## 6. Empirical Results

**Figure 1:**
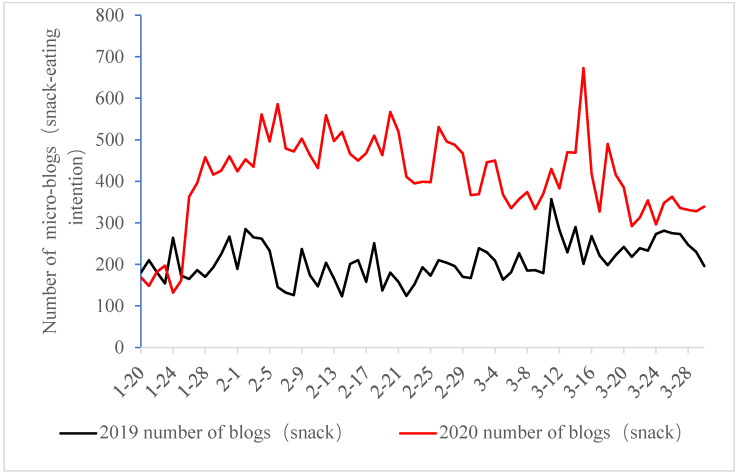
Day-to-day fluctuations in the number of snack-eating intention-associated microblogs.

**Figure 2:**
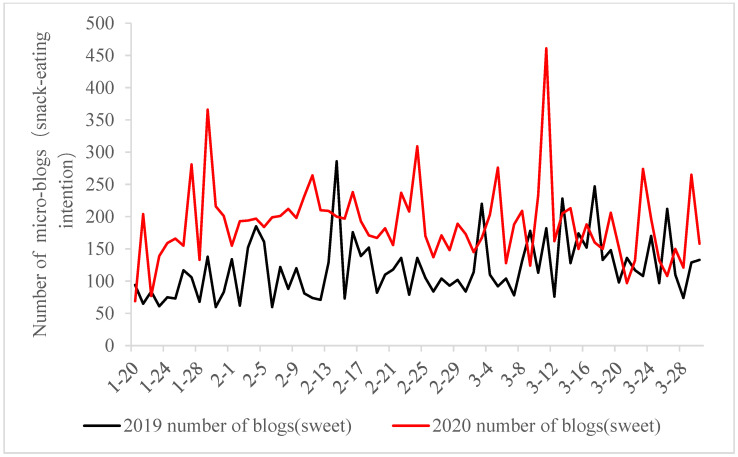
Day-to-day fluctuations in the number of sweet-eating intention-associated microblogs.

This study, which examines microblogs connected to sweet and snack intentions, reveals the increasing prevalence of the topics “want to eat snacks” and “want to eat sweets” during the COVID-19 pandemic in 2020. As shown in Figure 1, the number of microblogs related to snack-eating intention amounted to 29,598, much bigger than 14,714 during the same period in 2019. As shown in Figure 2, blogs that show sweet-eating motivation manifest the same trend as snack-eating, and the number during the pandemic period of 2020 is 13,194, higher than 8810 in the same period of 2019. In terms of snack-eating intention, the number of microblogs on snack-eating motivation continued to increase after 27 January 2020, as isolated management measures improved gradually in all Chinese provinces and cities. This trend was not seen at the same time last year. After that, it saw a slow decrease in foreign assaults at the end of February 2020.

### 6.1. Analysis of the Attitude Toward Eating Sweet and Snack

Utilizing Baidu’s AI platform, which integrates both an emotion dictionary and the method of machine learning, this study leveraged its deep learning capabilities and extensive real-sample datasets. The platform’s algorithms continuously refine the data, building a comprehensive emotional lexicon. For this research, microblogs analyzed using Baidu’s AI platform were evaluated, keeping only those with a confidence level above 80%. This approach enabled the calculation of the emotional proportions and sentiment scores of microblogs, with scores ranging from 0 (complete negative) to 1 (positive), as some examples illustrated in Table 2.

### 6.2. General Attitude Trend

**Figure 3:**
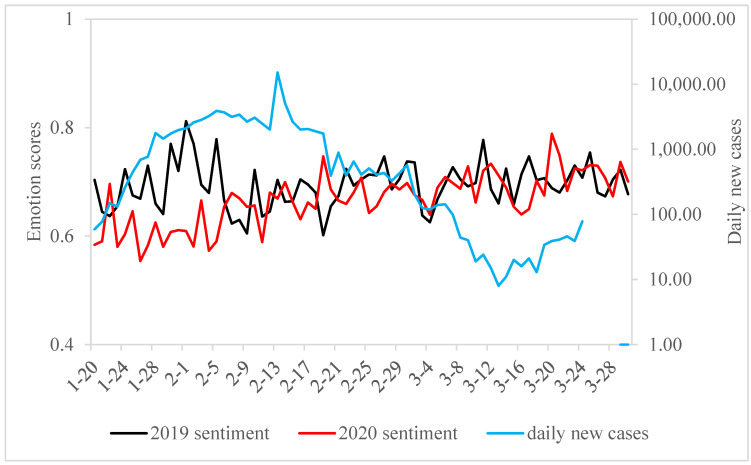
“Snack” blogs daily mean emotion scores and daily new cases.

**Figure 4:**
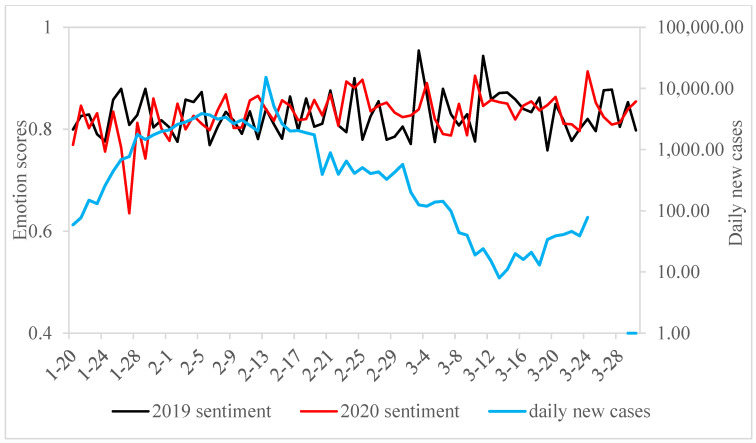
“Sweets” blogs daily mean emotion scores and daily new cases.

In this research, the emotional classification and filtering of microblog content were conducted using a confidence coefficient, focusing on microblogs from equivalent periods in 2019 and 2020. Table 3 demonstrates that a significant portion of microblog users possess a positive outlook towards eating snacks and sweets. Notably, the number of posts expressing the desire to eat these foods in 2020 substantially surpassed those in 2019. By 30 March, domestic case numbers in China had fallen to zero, with all new cases being from outside the country. Nevertheless, the sentiment towards consuming snacks reached its highest point on 20 March 2020, as depicted in Figure 3. In a similar vein, the emotional score relating to the consumption of sweets attained its maximum on 24 March 2020, as indicated in Figure 4.

## 7. Discussion

This research categorizes the process of compensatory consumption into three distinct stages: the anxiety phase, the escapism phase, and the compensatory phase, as outlined in the theory basement above. The conclusion aligns with the psychological phenomenon outlined in the literature review on compensatory consumption and emotional eating. We verify this phenomenon with real-time textual data from a novel perspective to demonstrate the intention of compensatory consumption.

### 7.1. Anxiety Phase

The anxiety phase of compensatory consumption intention in China took place between 20 January and 30 January 2020. As illustrated in Figure 3, the sentiment scores for snacks and sweets during this phase in 2020 were lower than the corresponding period in 2019, particularly for snacks. The emotional eating tendencies for snacks and sweets during this period were 0.60454 and 0.78673, respectively, indicating a relatively low level among the three stages. On 20 January, the National Health Commission of China announced the human-to-human transmission of COVID-19. However, since the epidemic had not yet broadly impacted the entire country, people did not feel an immediate danger. At this stage, the government’s exploration of the novel coronavirus was still in its initial phase, providing limited authoritative information, leading to a general lack of understanding about the virus and the pandemic among the public. Additionally, 25 January coincided with the Spring Festival, notably the Chinese New Year. During the Spring Festival holiday period (20–30 January), people usually return to their hometowns to reunite with family, but differing views on the pandemic among family members intensified concerns about the COVID-19 outbreak. Meanwhile, the youth and middle-aged groups, having access to various sources of information about the pandemic, likely had a better perception of the dangers and severity of COVID-19. In contrast, the elderly, due to a lack of exposure to information related to COVID-19, did not have a comprehensive understanding of the severity of the virus. Younger demographics, such as students, also exhibited different levels of awareness and concern. On 23 January, Wuhan, the city in Hubei Province where the earliest cases of COVID-19 were reported in China, went into lockdown, with all transportation leaving the city being suspended. Although the government did not explicitly restrict residents of other provinces and cities from going out at this stage, the majority of people, due to panic over the coronavirus, were reluctant to leave their homes. This led to disruptions in their daily social activities and normal life, consequently diminishing their sense of control over their lives.

Overall, this stage was the initial phase of the pandemic, where people’s understanding of the novel coronavirus was still insufficient. However, this might have led to significant panic and anxiety due to the limited information available, such as the number of deaths and the lockdown of Wuhan. This phenomenon aligns with the “take the best” heuristics, a concept that falls under Herbert Simon’s theory of bounded rationality [97]. As a result, people’s increased uncertainty about the development of the COVID-19 pandemic and their future lives made them more prone to experiencing higher levels of anxiety [98], and so people dared not go out or order desserts and snacks.

### 7.2. Escapism Phase

The escapism period roughly spanned from 31 January to 19 February. During this phase, people exhibited higher emotional eating tendencies towards snacks and sweets, with scores of 0.65329 and 0.83313, respectively, which were both higher than the previous period. During this time, most provinces and autonomous regions in China successively issued the first-level public security event response mechanism. The mandatory stay-at-home measures imposed by local governments led people to strongly desire a return to their daily activities. As the duration of home isolation extended, people began adapting to the perceived danger of the COVID-19 pandemic. Simultaneously, their eagerness to resume normal activities grew stronger, leading to a more pronounced inclination to escape from their previous anxieties and stress. Consequently, an increasing number of individuals sought alternative ways to relieve their feelings of pressure, gradually developing a desire for desserts and snacks, resulting in a further increase in their emotional eating tendencies.

### 7.3. Compensatory Phase

The compensatory period extended from 20 February to 30 March. During this phase, the emotional eating tendencies for snacks and sweets reached 0.68391 and 0.84064, respectively. Starting on 19 February, various provinces and regions across the country began to relax the mandatory first-level public security event response mechanism. People’s fear and anxiety about the pandemic gradually diminished during this period, although they remained cautious and were reluctant to visit public places. However, compared to the previous two periods, people were more inclined to be strongly motivated by compensatory consumption due to the previous feelings of suppression, anxiety, helplessness, and a sense of loss of freedom [15,99]. The emotional tendencies towards the emotional eating of snacks and sweets reached their peak during this stage. The lifting of the public safety response mechanism allowed people to engage in relatively freer activities, making emotional eating more accessible. However, most people are not consciously aware when they engage in emotional eating [100]. Therefore, Weibo posts during this stage about people’s eager desire for compensatory emotional eating are accurate.

## 8. Conclusions

This study has provided a comprehensive analysis of the impact of the COVID-19 pandemic on consumer compensatory eating intention, with a particular emphasis on the phenomenon of compensatory eating. Through the lens of the release theory and the theory of compensatory consumption, the research has successfully dissected the complex relationship between the pandemic’s psychological impact and the resultant changes in eating intention.

Our findings indicate that the COVID-19 pandemic, marked by unprecedented lockdowns and social distancing measures, significantly altered the psychological state of consumers, leading to an increased tendency towards emotional eating. This shift was particularly evident in the consumption of snacks and sweets, as revealed by the sentiment analysis of Sina Weibo posts. The data from 2020, compared to 2019, showed a substantial increase in posts expressing a desire for compensatory consumption of these food items. This study identified three distinct stages in consumer behavior during the pandemic: the anxiety period, the escapism period, and the compensatory period. Each stage corresponds with specific changes in the public’s emotional state and compensatory eating patterns. The anxiety period saw a subdued response, likely due to the initial shock and uncertainty of the pandemic. During the escapism period, as the reality of prolonged isolation set in, there was a noticeable increase in emotional eating tendencies. Finally, the compensatory period, marked by a gradual relaxation of lockdown measures, saw the highest levels of compensatory eating, reflecting a release from previously suppressed desires and a bid to regain a sense of normalcy and control. Moreover, the innovative approach of utilizing social media sentiment analysis provided valuable insights into the real-time emotional states of consumers, offering a novel perspective in understanding the dynamics of compensatory consumption. This approach highlighted the significance of digital platforms in gauging public sentiment and behavior patterns, especially during global crises.

The implications of this study are far-reaching and can offer crucial insights for policymakers and health professionals. Understanding the psychological impact of pandemics on eating behaviors can inform better strategies for public health messaging and interventions. For instance, governments are supposed to give relevant food intake advice to remind the public to control the amount of junk food during a public emergency period. Moreover, paying attention to the reflection of public compensatory consumption intention on social media can help governments learn about public mental health quickly during a public emergency. Thus, governments can better take action to intervene in consumption and the operation of enterprises. For marketers and the food industry, these insights into consumer behavior can guide more empathetic and effective marketing strategies during and after such global crises. This can provide a theoretical basement for releasing food advertisements in public emergency periods. In conclusion, the COVID-19 pandemic has underscored the intricate interplay between psychological stressors and consumer behavior, particularly in the realm of compensatory eating. As the world navigates similar challenges in the future, the insights garnered from this study can serve as a valuable resource in anticipating and managing the implications of such events on consumer behavior.

This study, while providing valuable insights into the relationship between the COVID-19 pandemic and compensatory eating behaviors, is not without its limitations. The research primarily focuses on data from China, specifically utilizing Sina Weibo posts. This geographical focus limits the generalizability of the findings to other cultural and social contexts. Integrating additional psychological and sociological theories in future research could offer a more nuanced and comprehensive understanding of the dynamics underlying compensatory consumption behaviors. Future research could also benefit from integrating other psychological and sociological theories to provide a more nuanced understanding of the dynamics behind compensatory consumption. Conducting longitudinal studies such as natural disasters and other pandemics would help in understanding how compensatory eating behaviors evolve over time, particularly as the world recovers from the COVID-19 pandemic.

## Figures and Tables

**Table 1:** Weibo posts data sample.

Weibo ID	Weibo Micro-Blogs Content	Time	Location
5760074428	I want sweets, really want dessert, only dessert can briefly console me. If you have any other requests, feel free to let me know.	21 January 2019 01:44	Hunan Province
5553759555	I really want to eat sweets!!!	27 February 2019 02:06	Fujian Province
6576196777	When I’m feeling low, I just go crazily for snacks! I mean, gotta fill either the heart or the stomach, right?	12 January 2019 19:51	Hunan Province
2860272755	I really want some snacks, oh, I’m so hungry, and I’m so tempted. Is there anyone hungrier or more tempted than me? Nope.	14 March 2019 16:56	Beijing
5863797472	Unable to resist the craving for sweets, I desperately want to eat sweets, taking matters into my own hands. Checked the refrigerator and found butter and whipping cream. Going to see what I can whip up to eat.	1 February 2020 09:05	Zhejiang Province
6723612579	I’ve never felt such an urgent need to visit the supermarket, nor have I ever wanted to eat snacks and hot pot so intensely. The way I am now, I truly feel like I’m going crazy from being cooped up.	29 January 2020 00:06	Jiangsu Province
3094077094	Really want to eat some sweets right now. Lately, I’ve been in the mood for something sugary. Can’t wait for this pandemic to be over so I can hit up a dessert spot.	18 February 2020 22:36	Henan Province
6189833227	I really want to eat some junk snacks. All I’ve got at home is chocolate, and it’s just not hitting the spot for me.	6 February 2020 15:00	Hebei Province

**Table 2:** Emotion analysis sample of eating attitude.

Sina Weibo Micro-Blogs Content	Confidence	Positive Emotion	Negative Emotion	Emotion Classification	Emotion Scores
I really, really want to eat chocolate, thinking of devouring all my chocolates. I also crave half-cooked cheese, cake, and milk tea. I used to be someone who didn’t like sweets at all, but life has been too bitter lately. Life is tough, and I need a little sweetness.	0.99185	0.99633	0.00367	Positive	0.99
OMG, after waking up, my craving for snacks has reached its peak. I also want to eat Beijing roast duck, have rice noodle rolls, drink milk tea, and eat ice cream!!!!	0.960622	0.98228	0.01772	Positive	0.98
Feeling quite bored, but not in the mood for watching shows, unable to focus on reading books, not interested in snacks, and even scrolling through Weibo isn’t bringing up any new content. I feel like it’s not true idleness, but genuine laziness.	0.99998	0.00001	0.99999	Negative	0.00
Staying at home, not in the mood to eat anything or watch anything. Just sitting in front of the TV, munching on snacks to pass the time. [sick]	0.99549	0.00203	0.99797	Negative	0.002
Before the Chinese New Year, I didn’t stock up on snacks because I usually don’t eat them much. However, now I really, really, really want to eat packaged potato chips but I’m afraid to go out. [cry][cry]	0.84534	0.50735	0.49265	Neutral	0.50
I slept for twenty minutes, just woke up, and have been scrolling on my phone ever since. I really, really, super-duper want to eat hot pot, spicy hot pot, barbecue, desserts, and all kinds of junk food from McDonald’s and KFC. The early sleep and wake-up plan failed once again. Suddenly, I really want to go to work and get back to a regular schedule. [love for you]	0.82916	0.52188	0.47812	Neutral	0.52

**Table 3:** Statistical table of categories emotions (snacks and sweets).

	Positive	Negative	Neutrality	Total
The number of microblogs (snack) 2020	19,450	9173	975	29,598
The number of microblogs (snack) 2019	8864	5331	519	14,714
The number of microblogs (sweet) 2020	10,894	2010	290	13,414
The number of microblogs (sweet) 2019	7372	1155	181	8514

## Data Availability

The original contributions presented in the study are included in the article, further inquiries can be directed to the corresponding author.
